# Peculiarities of the Structure of Au-TiO_2_ and Au-WO_3_ Plasmonic Nanocomposites

**DOI:** 10.3390/ma16206809

**Published:** 2023-10-22

**Authors:** Yerulan Sagidolda, Saule Yergaliyeva, Zhandos Tolepov, Guzal Ismailova, Bakytzhan Orynbay, Renata Nemkayeva, Oleg Prikhodko, Svetlana Peshaya, Suyumbika Maksimova, Nazim Guseinov, Yerzhan Mukhametkarimov

**Affiliations:** 1 Department of Physics and Technology, Al-Farabi Kazakh National University, Al-Farabi av. 71, Almaty 050040, Kazakhstan; erulan.sagidolda@kaznu.kz (Y.S.); oleg.prikhodko@kaznu.kz (O.P.); svetlana.mikhailova@kaznu.kz (S.P.);; 2 National Nanotechnology Laboratory of Open Type, Al-Farabi av. 71/23, Almaty 050040, Kazakhstan

**Keywords:** gold nanoparticles, oxide thin films, radio frequency magnetron sputtering, Raman spectroscopy, plasmonics

## Abstract

As nanotechnology continues to advance, the study of nanocomposites and their unique properties is at the forefront of research. There are still various blank spots in understanding the behavior of such composite materials, especially regarding plasmonic effects like localized surface plasmon resonance (LSPR) which is essential for developing advanced nanotechnologies. In this work, we explore the structural properties of composite thin films consisting of oxide matrices and gold nanoparticles (Au NPs), which were prepared by radio-frequency magnetron sputtering. Titanium dioxide (TiO_2_) and tungsten trioxide (WO_3_) were chosen as the host matrices of the composites. Such composite thin films owing to the presence of Au NPs demonstrate the LSPR phenomenon in the visible region. It is shown, that spectroscopic study, in particular, Raman spectroscopy can reveal peculiar features of structures of such composite systems due to LSPR and photoluminescence (PL) of Au NPs in the visible spectrum. In particular, defect peaks of TiO_2_ (700–720 cm^−1^) or WO_3_ (935 cm^−1^) in Raman spectra can be clearly observed when the samples are illuminated with a 633 nm excitation laser. Excitation with 532 nm leads to a decrease in the intensity of the defect peak, which totally disappears at 473 nm excitation. Such dependences of the defect peaks on excitation laser wavelength are probably related to the polarization of the matrix’s defective regions close to the interface with gold NPs.

## 1. Introduction

Despite the high cost, gold (Au) nanoparticles (NPs) still attract tremendous attention of science owing to their unique plasmonic properties and excellent chemical stability. Similar to silver (Ag) NPs, Au NPs demonstrate a localized surface plasmon resonance (LSPR) phenomenon [1,2]. The last takes place when the frequency of incident light coincides with the frequency of plasmon oscillation, the collective oscillation of free electrons in metal NPs. Important, that the resonance frequency depends on the sizes, shapes, and surrounding medium of NPs which opens the opportunity to tune the LSPR properties of metal NPs. For spherical Au NPs, the resonance frequency is within the visible light range.

Modern applications of Au NPs related to the LSPR phenomenon are focused on its three inherent features. The point is that when a nanoparticle is irradiated at the resonant frequency the strong localized electric field is created at its surface [3,4]. The metal NPs demonstrate the light-harvesting properties. At the same time, the cross-section of light-matter interaction becomes larger than the real geometrical sizes of NPs [1]. Therefore, macro-molecules located near the surface of Au NP can be polarized under a strong electrical field. This phenomenon became the basis for the detection of single molecules in the Surface Enhanced Raman Spectroscopy (SERS) technique [4]. On the other hand, the dependence of the LSPR peak on the surrounding medium makes it possible to create LSPR sensors [5,6], which can detect changes in the refractive index or charges of the medium molecules. A survey of recent publications indicates the growing interest in thermo-optical (thermoplasmonics) properties of Au NPs especially for medical purposes and photocatalysis [7,8,9]. Finally, processes of hot electron injection from NPs irradiated at the resonance frequency to the surrounded medium are crucial factors that improve various properties of semiconducting materials, luminescent centers, etc. It is clear that the combination of metal NPs with wide-gap semiconductors increases the photocatalytic performance of the latter. Metal NPs reduce the recombination rate of non-equilibrium charge carriers of the semiconductor, and at the same time significantly increase the light-harvesting properties of a wide-gap semiconductor in the visible range of radiation.

The prominent representatives of wide-gap semiconductors are titanium dioxide (TiO_2_) and tungsten trioxide (WO_3_) which are widely used in various applications today. In particular, electrodes based on TiO_2_ are appealing for water-splitting processes, photo-catalytic air and water purification as well as solar cell production [10,11,12]. Much research has demonstrated an enhancement of photocatalytic properties of TiO_2_ both in the ultraviolet (UV) and visible ranges due to the charge transfer processes at the “oxide/metal NPs” interfaces in the systems both with periodically arranged plasmonic nanostructures and randomly distributed plasmon nanoparticles embedded in oxide matrix [13,14,15,16,17,18]. Tungsten trioxide WO_3_ is generally used as the electrochromic coating for smart windows due to its ability to change color depending on its stoichiometry [19,20]. Recently, there has been a growing trend of studying the photo-induced enhancement of Raman signal (PIERS) using composites based on the considered oxides with noble metal nanoparticles [21,22].

In most cases, such composites are synthesized by cost-effective aqueous chemical methods [23,24,25,26]. The less popular synthesis methods are traditional physical vapor deposition (PVD) techniques such as magnetron sputtering, laser ablation, etc. [27,28,29,30,31]. Depending on the synthesis method, the prepared electrodes have different photocatalytic and photo electrochromic efficiencies due to the different porosity and crystallite sizes of the oxide semiconductors. The presence of structural defects in the composite is also important [22]. Therefore, in this work, using Raman spectroscopy we have investigated the structural properties of Au-TiO_2_ and Au-WO_3_ composites obtained by the RF magnetron sputtering method.

In general, spectroscopic studies, especially Raman spectroscopy, of plasmonic composite systems based on a dielectric matrix and noble metal NPs exhibit peculiar features due to localized surface plasmon resonance phenomena and photoluminescence in the visible spectrum. Therefore, the signal collected by the Raman spectrometer can depend on the excitation laser wavelength. The main goal of this contribution is to demonstrate the peculiarities of the Raman spectroscopy analysis of plasmonic composite systems.

## 2. Materials and Methods

Pure (TiO_2_, WO_3_) and composite (Au-TiO_2_, Au-WO_3_) thin films were deposited onto the UV transparent quartz (10 × 20 mm^2^), KBr salt and Si (10 × 10 mm^2^, n-type, (100), 5–10 Ohm) substrates by radio-frequency (RF, 13.56 MHz) magnetron (Onyx 3′′, Angstrom Science, Duquesne, PA, USA) sputtering method. Circular 3-inch in diameter rutile (4 N) and blue-colored tungsten oxide (4 N) targets were used as sputtering sources. To obtain composite thin films, 2 Au pellets (1 × 5 mm^2^) were placed symmetrically onto the erosion zone of sputtering targets. The sputtering was carried out at a constant 0.5 Pa working pressure using high-purity argon (Ar, 99.999%) gas with a flow rate of ~50–60 sccm without external substrate heating. The deposition time for all samples was 1 h. Before deposition, the vacuum chamber was evacuated to 10^−4^ Pa. The substrate holder rotated at a speed of 12–13 rpm. The power of the RF source was set to 100 W for all depositions. The distance between the targets and the substrate holder was 80 mm, and between electrodes was fixed at 5 mm. The detailed geometrical parameters can be found in work ref. [32,33].

As-deposited films were annealed in the air atmosphere for 20 min. The annealing temperature was 450 °C for TiO_2_ and Au-TiO_2_ and 550 °C for WO_3_ and Au-WO_3_. The cooling was natural. These temperatures are typical calcination temperatures of the corresponding matrices. The short annealing time was used to avoid the excess coalescence of the metal nanoparticles. The longer annealing may lead to the increase of Au NP size on the surface of the composite thin films.

Characterization of the prepared thin films was carried out using standard measurement techniques such as scanning and transmission electron microscopy (SEM, TEM), spectrophotometry, X-ray diffraction analysis, and Raman spectroscopy. The elemental compositions of the films were controlled by energy-dispersive analysis using an EDAX detector on a scanning electron microscope (Quanta 3D 200i, FEI Company, Brno, Czech Republic). The thicknesses of the films were controlled by a quartz crystal monitor and verified by scanning the cross-section of the films on Si substrates using SEM. 

The presence of isolated gold NPs in composite thin films was confirmed by TEM (JEM-1400 Plus, JEOL Ltd., Tokyo, Japan) analysis and by detecting the plasmonic resonance absorption peak on the absorbance spectra acquired using a spectrophotometer (Shimadzu 3600, Shimadzu Co., Ltd., Kyoto, Japan) in the spectral range from 250 to 1200 nm with a slit of 1 nm. TEM measurements were carried out in the bright field mode using an accelerating voltage of 120 keV. 

The structural properties of the films were characterized using an X-ray analytical system (Rigaku Miniflex, Rigaku Co., Ltd., Tokyo, Japan) with a CuKα monochromator and Raman spectroscopy (Raman spectrometer based on Solver Spectrum, NT-MDT Co., Ltd., Zelenograd, Russia). Scan speed and step width during the XRD measurements were 10 deg./min and 0.02 deg., respectively. Raman signals were recorded using 473, 532, and 633 nm excitation lasers in the range from 50 to 1500 cm^−1^ and an exposure time of 100 s. Spectra of photoluminescence was acquired using the same Raman spectrometer with a wide-range diffraction grating and a 473 nm laser. 

The surface morphology of the synthesized films was studied using atomic force microscopy (AFM) on the Solver Spectrum (NT-MDT Co., Ltd., Zelenograd, Russia) instrument in semi-contact mode; the scan area was 3 × 3 µm for all samples.

For SERS measurements, annealed films of TiO_2_, Ag-TiO_2_, Ag/Au-TiO_2_, and Au-TiO_2_ deposited on quartz substrates were immersed in 10^−5^ M aqueous solution of Methylene blue (MB) for 1 h and then were dried in air at room temperature. SERS spectra were acquired with a 633 nm excitation laser.

## 3. Results

### 3.1. Characterization of Au-TiO_2_ Thin Films

**Figure 1:**
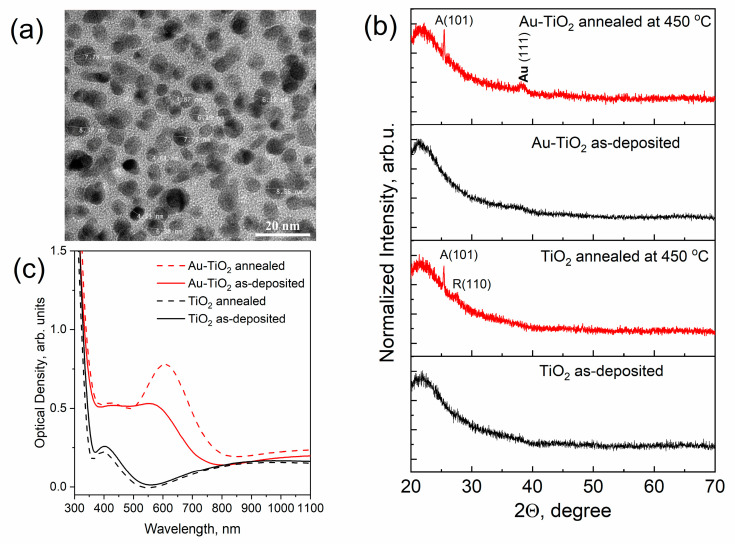
TEM image of Au-TiO_2_ thin film (**a**) and the results of a comparative study of pure TiO_2_ and composite Au-TiO_2_ thin films by means of XRD analysis (**b**), and optical density spectra (**c**).

RF sputtering of a combined target consisting of a ceramic wafer (rutile, TiO_2_) and gold pellets made it possible to obtain composite thin films with metal nanoparticles. Figure 1a shows typical TEM images of as-deposited TiO_2_ composite film with an Au concentration of ~5 at.% and a thickness of sample of 125 ± 5 nm. As can be seen in the figure, dark spots correspond to gold metal nanoparticles. The results of the determination of the elemental composition of the films are provided in Figure S1 of Supplementary Information (SI). XRD analysis shows that the obtained pure and composite TiO_2_ thin films have an amorphous structure (See Figure 1b, black curves). The presence of isolated nanoparticles in composite thin films contributed to the appearance of the effect of localized surface plasmon resonance of light absorption in the visible range. The solid lines in Figure 1c correspond to the optical density spectra of pure TiO_2_ and Au-TiO_2_ thin films. As can be seen in the optical spectra of the Au-TiO_2_ thin film possible plasmonic resonance absorption peak is suppressed by the interference peak and as a result, we observe a flat step-like shoulder in the range of 400–550 nm. 

**Figure 2:**
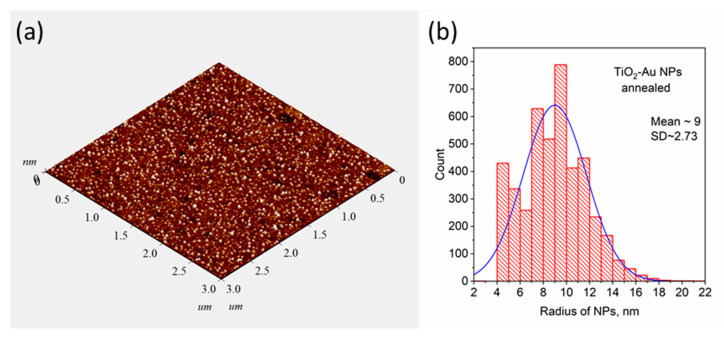
AFM image of annealed Au-TiO_2_ thin film (**a**) and the results of determination of particle size distribution (**b**).

The situation slightly changes after the thermal treatment of the films. Annealing of TiO_2_ thin films (450 °C) led to the crystallization of TiO_2_ matrix. Red lines in Figure 1b correspond to the XRD patterns of TiO_2_ and Au-TiO_2_ thin films in which the main diffraction peak of the anatase structure (101) can be observed. The intensity of the XRD signal is low due to the small thicknesses of the investigated TiO_2_-based samples. At the same time high temperature stimulates the diffusion of metal ions and coalescence of the Au NPs on the surface of the TiO_2_ film which increases the efficiency of LSPR (see Figure 1c). AFM studies of the morphology of the films before and after annealing showed an increase in the number of Au NPs on the surface of the film. In Figure 2a AFM image of the morphology of annealed Au-TiO_2_ thin film is shown. The mean size of Au NPs on the surface according to the defined distribution (Figure 2b) is about 18 nm. The AFM images of the rest samples and contrast AFM images used for the size distribution analysis are represented in Figures S2 and S3 of SI. The optical density spectrum of the annealed Au-TiO_2_ composite thin film (red dashed line in Figure 1c) is characterized by the distinct plasmonic absorption peak at the wavelength of 605–610 nm.

### 3.2. Characterization of Au-WO_3_ Thin Films

**Figure 3:**
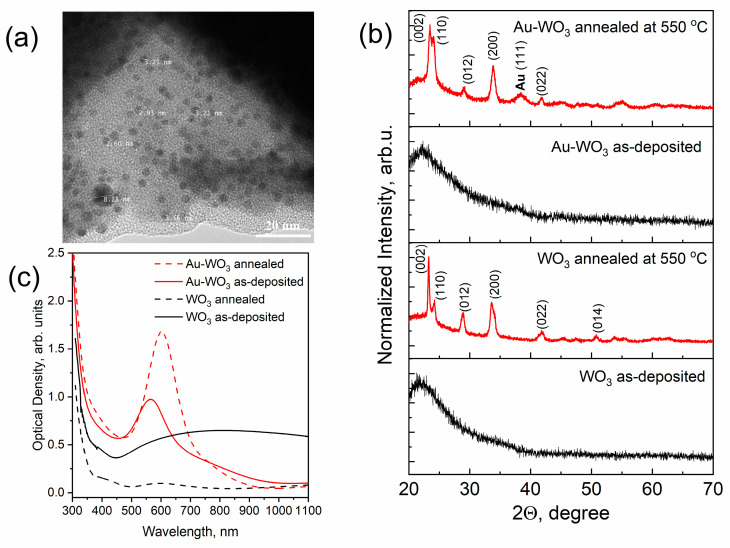
TEM image of as-deposited Au-WO_3_ thin film (**a**) and the results of a comparative study of pure WO_3_ and composite Au-WO_3_ thin films by means of XRD analysis (**b**), and optical density spectra (**c**).

**Figure 4:**
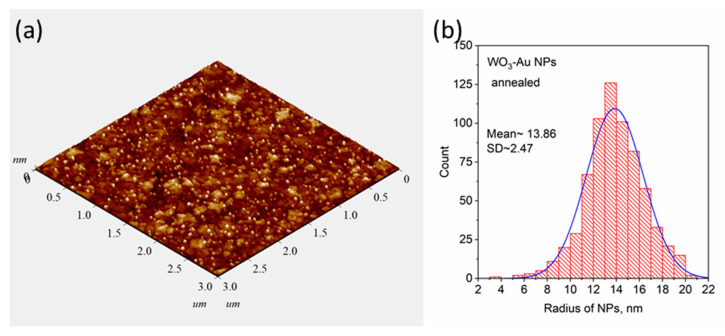
AFM image of annealed Au-WO_3_ thin film (**a**) and the results of determination of particle size distribution (**b**).

Similar studies were carried out for pure and composite WO_3_ films, which were obtained using the blue-colored WO_3_ sputtered target with Au pellets. Figure 3a shows a typical TEM image of an as-deposited Au-WO_3_ composite thin film with an Au concentration of about 3–4 at.%. As can be seen from the image there are also dark spots corresponding to Au NPs observed. In contrast to Au-TiO_2_, here, the mean size of spots is smaller in volume. This can be explained by the fact that the sputtering rate of the WO_3_ target is higher than that of the TiO_2_ one. Thus, at the same deposition condition and duration, the Au-WO_3_ composite thin film has a larger thickness (~190 nm). In Figure 3b XRD patterns of the WO_3_ and Au-WO_3_ films before and after annealing (550 °C) are presented. According to these results, one can conclude that the WO_3_ matrix rearranged from an amorphous to a monoclinic phase after annealing. The isolated AuNPs almost do not influence the structure of the WO_3_ matrix. Figure 3c shows the optical density spectra of pure WO_3_ and composite Au-WO_3_ thin films. As can be seen, the maximum LSPR peak of Au-WO_3_ thin film shifts from 570 to 603 nm after annealing. The spectra of WO_3_ indicate that as-deposited thin films have poor transparency due to the blue coloration and lack of stoichiometry. Annealing leads to obtaining bleached WO_3_ thin films with excellent transparency. Similar to the previous case, the AFM image of the annealed Au-WO_3_ film shown in Figure 4a demonstrates the presence of nanoparticles on the surface, but in contrast with TiO_2_, the WO_3_ matrix has larger grain sizes. The results of the size distribution analysis showed that the mean diameter of the nanoparticles on the surface is about 27 nm (Figure 4b). The rest topographical scans and contrast AFM images used for size distribution can be found in Figures S2 and S3 in the SI Section. 

### 3.3. Raman Spectroscopy of Composite Thin Films

**Figure 5:**
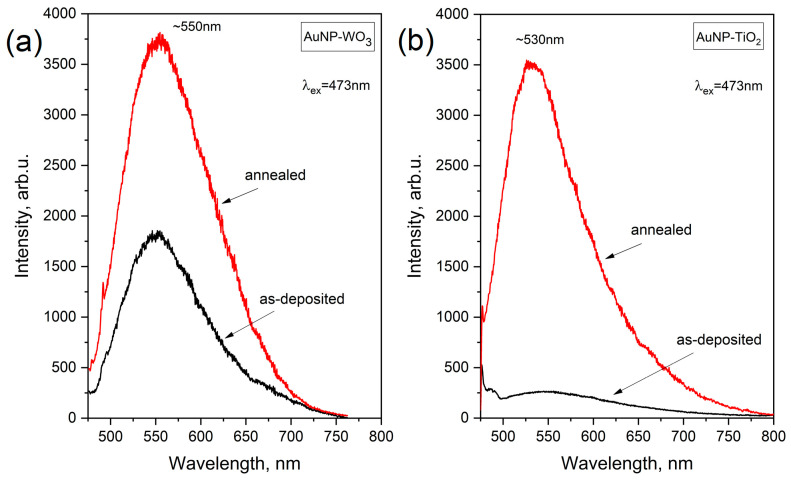
PL spectra of Au-WO_3_ (**a**) and Au-TiO_2_ (**b**) films before and after annealing.

When studying plasmonic composite systems by Raman spectroscopy, there are several challenges and intricacies due to LSPR phenomena. The presence of plasmonic NPs can drive strong enhancements or quenching of Raman signals due to the interaction between the localized electromagnetic fields and the material surrounding plasmonic NPs. In addition, it is known that gold and gold nanoparticles owing to interband electronic transitions can emit photons with corresponding energy of ~2.3 eV [34]. This in turn impacts the acquired signal of Raman scattering. Figure 5 demonstrates this effect showing the PL spectra of the Au NPs-embedded films before and after annealing. It can be seen that the films exhibit wide-range PL signal with a maximum at ~530 nm (2.34 eV) and ~550 nm (2.25 eV), for TiO_2_ and WO_3_, respectively. 

**Figure 6:**
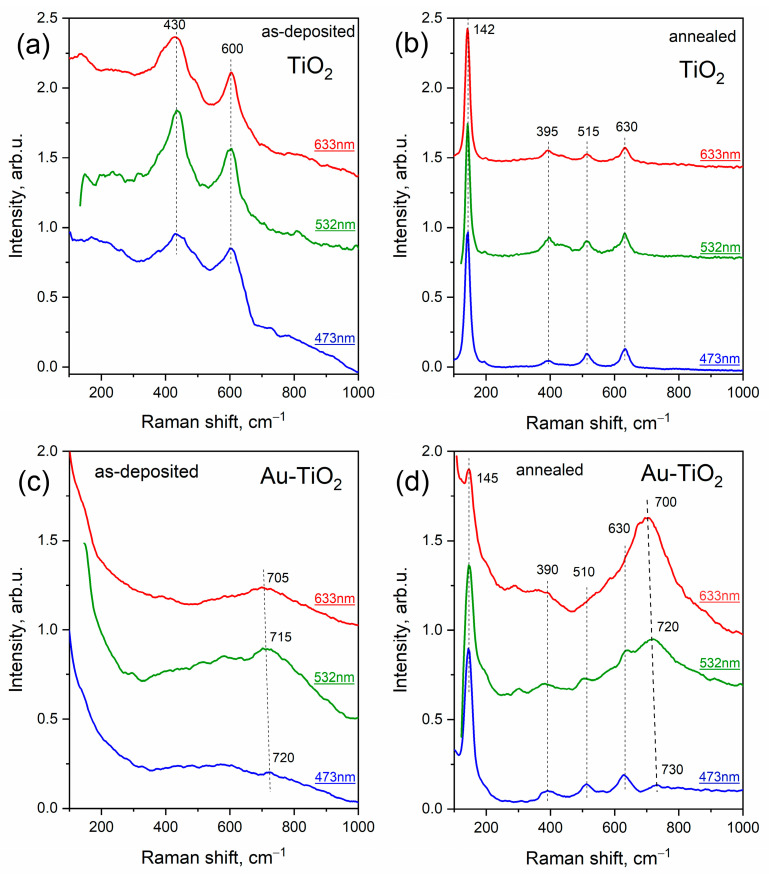
Raman spectra of pure TiO_2_ and composite Au-TiO_2_ thin films before (**a**,**c**) and after (**b**,**d**) annealing.

Thus, for comprehensive Raman studies, three excitation lasers with wavelengths of 473, 532, and 633 nm were used. Figure 6 shows Raman spectra of pure and Au-TiO_2_ composite thin films acquired at different excitation wavelengths. Interestingly, pure as-deposited TiO_2_ film demonstrates an amorphous structure with typical broad peaks at about 430 and 600 cm^−1^, while composite as-deposited Au-TiO_2_ film does not demonstrate any distinct peak possibly indicating a more disordered structure (Figure 6a,c). However, for Au-TiO_2_ there is a broad band in the region of 700–720 cm^−1^, which according to numerous experiments is typical (characteristic) for this kind of system. In ref. [35] this peak was assigned to the defective structure of TiO_2_, particularly to titanates of different (non-noble) metals. 

According to performed XRD analysis, annealing of the synthesized films leads to crystallization of the matrix, which was also confirmed by the results of Raman characterization. Pure TiO_2_ after annealing demonstrates typical Raman modes E_g_ (142 cm^−1^, 630 cm^−1^), B_1g_ (395 cm^−1^), A_1g_ (515 cm^−1^) corresponding to anatase structure (see Figure 6b). Raman spectra of annealed Au-TiO_2_ depicted in Figure 6d can also be assigned to the anatase phase, because the most prominent peak at 145 cm^−1^ corresponding to E_g_ vibrational mode serves as an indicator of this phase, while the small shift of the peaks from typical positions is apparently due to the presence of metal nanoparticles. Interestingly, the broad peak at 700–720 cm^−1^ remained after treatment. However, the dependence of intensity and position of this peak on the excitation wavelength became considerable. As can be seen from Figure 6d, the intensity of this peak increases with increasing laser wavelength, while the position still shifts towards the low-frequency region. 

The same pattern was observed for the system of Au-WO_3_, where the presumably defect peak demonstrates strong dependence on the laser wavelength. 

**Figure 7:**
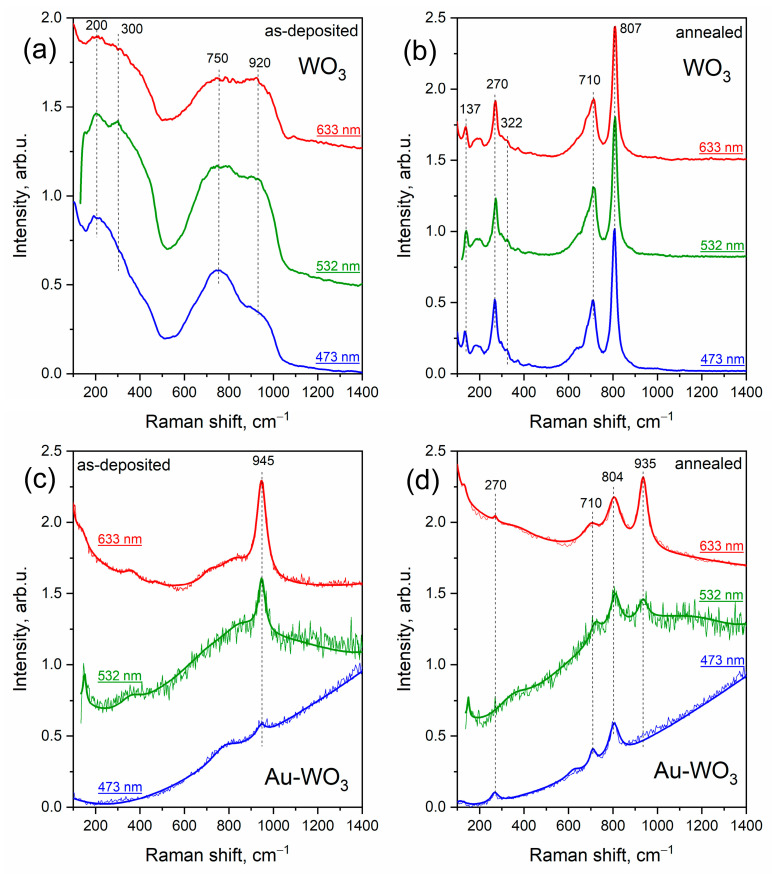
Raman spectra of pure WO_3_ and composite Au-WO_3_ thin films before (**a**,**c**) and after (**b**,**d**) annealing.

Raman spectra of as-deposited WO_3_ and Au-WO_3_ films acquired using different excitation wavelengths are presented in Figure 7a,c. Similarly to TiO_2_, pure WO_3_ film demonstrates a typical amorphous structure with broad bands at about 200, 300, 750, and 920 cm^−1^. In the case of Au-containing as-deposited WO_3_ film, a signal of amorphous WO_3_ structure is suppressed by a peak at 945 cm^−1^ and a rather strong PL trend. In ref. [36] the rise of the peak at about 920–950 cm^−1^ is assigned to the formation of the sub-stoichiometric structure W_18_O_49_, which is quite possible for such systems. However, in our case, XRD analysis did not reveal this phase. 

As stated above, post-synthesis heat treatment led to crystallization of the matrix, and Raman spectra of the annealed pure WO_3_ film show vibrational modes corresponding to monoclinic tungsten oxide (WO_3_) (see Figure 7b,d). A peak at 270 cm^−1^ corresponds to low-frequency O-W-O vibrational mode, while high-frequency peaks at 807 cm^−1^ and 710 cm^−1^ are due to symmetric and asymmetric stretching vibrations of bridging oxygen. Au-containing WO_3_ film also demonstrates crystalline features after annealing, however typical peaks of monoclinic tungsten oxide are slightly shifted and suppressed by the PL trend. Similarly to Au-TiO_2_ film, here we observe a strong dependence of defect peak at ~940 cm^−1^ on the excitation wavelength. Under 473 nm excitation, the spectrum fully coincides with that of pure monoclinic tungsten oxide WO_3_ along with an increasing PL trend. While under excitation with a red laser at 633 nm, AuNPs-embedded film demonstrates an intense defect peak at 935 cm^−1^.

## 4. Discussion

These findings are important and interesting, since using different excitations one can observe a pure crystalline matrix or reveal considerable defects in the same composite films. We suppose that the appearance of the peaks at 700–720 cm^−1^ and 935 cm^−1^ for TiO_2_ and WO_3_ matrices, respectively, is induced by the polarization (due to the charge transfer) of the matrix’s defective regions close to the interface with gold NPs. When the considered systems are excited by short-wavelength excitation (473 nm), a radiative decay occurring in excited nanoparticles (see Figure 5) weakens both the local field near the particles and charge transfer processes at the metal-oxide interface. So, under this condition, the defect peak of the considered matrices is not observed in the Raman spectrum. 

On the other hand, the energy of the red laser (1.96 eV) is close to the energy of LSPR of gold nanoparticles, in which excited electrons undergo plasmonic decay due to hot electron injection into the surrounding matrix. The processes of interface recharge were investigated in ref. [37], where using the Kelvin probe force microscopy (KPFM) the changes in the surface potential were measured under the different light wavelengths. This process is able to form a depletion region on the metal-oxide interface or polarization of areas of the matrix neighboring with metallic NP. Polarization processes can be induced by the enhancement of the local field near the LSPR frequency on the metal-oxide interface that significantly affects the processes of charging of the interface area. Generally, the local field inside the nanoparticle can be expressed as:

(1) $$E_{in}=\frac{3\;\varepsilon_{h}\;}{\varepsilon \left(\omega \right)+2\;\varepsilon_{h}\;}E_{0}$$

where $E_{0}$—field of incident wave, $\varepsilon_{h}$—dielectric constant of the matrix, $\varepsilon \left(\omega \right)$—a complex dielectric function of metal nanoparticles. From this equation, it is seen that near-field enhancement occurs when $\varepsilon \left(\omega \right)+2\;\varepsilon_{h}\to 0$, or when the real part of the dielectric constant fulfills the condition $\varepsilon^{\prime }\left(\omega \right)\sim -2\;\varepsilon_{h}$, while the imaginary part $\varepsilon^{\prime \prime }\left(\omega \right)$ tends to the smallest value. Figure S4 (SI) shows the plot of real and imaginary parts of the dielectric function of bulk gold and silver taken from Johnson et al. [38]. As can be seen, unlike silver, gold has a narrow spectral range in which $\varepsilon^{\prime \prime }\left(\omega \right)$ does not exceed a value of 2–3. Therefore, SERS substrates based on gold nanoparticles effectively enhance the Raman signal under red and near-IR laser excitation. Note that, silver nanoparticles can enhance the Raman signal (SERS) in the entire visible range and even in the near-IR. 

**Figure 8:**
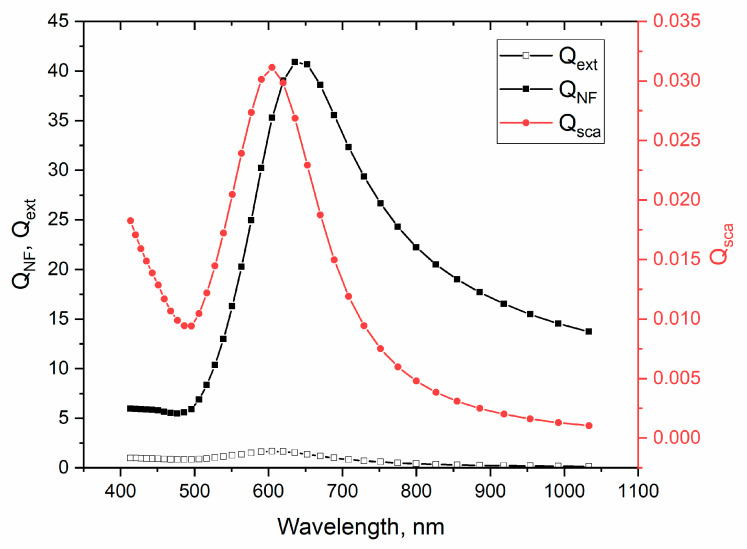
Spectral dependences of far ($Q_{sca}$), near ($Q_{NF}$) field scattering, and extinction ($Q_{ext}$) efficiencies.

Using the classical theory of light scattering it is possible to estimate the efficiency of light scattering in the near field [3]. Figure 8 shows the calculated efficiencies of extinction ($Q_{ext})$, scattering in the far field ($Q_{sca}$), and in the near field ($Q_{NF}$) which were obtained according to the following equations:

(2) $$Q_{ext}=\frac{2}{{\left(ka\right)}^{2}}\sum_{n=1}^{\infty }\left(2n+1\right)Re\left(a_{n}+b_{n}\right),$$

(3) $$Q_{sca}=\frac{2}{{\left(ka\right)}^{2}}\sum_{n=1}^{\infty }\left(2n+1\right)\left({\left|a_{n}\right|}^{2}+{\left|b_{n}\right|}^{2}\right)$$

(4) $$Q_{NF}=2\sum_{n=1}^{\infty }\left({\left|a_{n}\right|}^{2}(\left(n+1\right){\left|h_{n-1}^{\left(2\right)}\left(ka\right)\right|}^{2}+n{\left|h_{n+1}^{\left(2\right)}\left(ka\right)\right|}^{2})+(2n+1){\left|b_{n}\right|}^{2}{\left|h_{n}^{\left(2\right)}\left(ka\right)\right|}^{2}\right).$$

Here, $a_{n}$ and $b_{n}$—the Mie coefficients, $h_{n}^{\left(2\right)}\left(ka\right)$ second kind Hankel function, $k=2\pi n/\lambda_{o}$ is a wavenumber, and a denotes the radius of NP. These expressions were solved using the Wolfram Mathematica 11 software package. For the calculations, the value of the refractive index (RI) of the medium was chosen equal to 2.1, which is slightly lower than the typical RI values of TiO_2_ and WO_3_. The reason for this is the presence of the partially open sides of NPs on the surface of the composite films. The dielectric function of Au NPs was extracted from [38] and takes into account the size effects. Calculations of the efficiencies also take into account the size distribution of Au NPs according to defined normal distribution parameters (Figure 2b). It follows from the figure that the maximum conversion of the incident intensity into near-field scattering ($Q_{NF}$) occurs at 635 nm and the minimum is at wavelengths below 500 nm. This simple analytical estimation further supports the proposed explanation. Nevertheless, in this work, for confirmation of the above hypothesis, two additional studies were carried out.

In the first case, we have considered systems of ultra-thin (<10 nm) TiO_2_ and WO_3_ films deposited on Au NPs, obtained by annealing of thermally evaporated gold thin films. Despite the extremely low thickness of the oxide films, it was possible to study the dependence of their main Raman peaks on the excitation wavelength. The Raman spectra of such systems presented in Figure S5 of SI demonstrate similar trends. Such effects will probably be observed for core-shell NPs with Au core as well.

In the second case, we investigated the behavior of similar thin film composite systems containing silver (Ag) and gold-silver (Ag/Au) alloy nanoparticles. Such composite thin films were described in our previous works [32,33]. The Raman spectra of composite thin films with Ag NPs (see Figure S7 of SI) demonstrate a defect peak regardless of the wavelength of the excitation laser (473 nm, 633 nm) due to the high value of interband transition energy (4 eV) of Ag NPs and enhanced near-field in the entire considered spectral range. Bimetallic NPs such as Ag/Au alloy demonstrate the interband transition energy in the range between that of Au and Ag NPs. This, in particular, is confirmed by the PL spectra of Ag/Au-TiO_2_ and Ag/Au-WO_3_ thin films shown in Figure S6 of SI. Raman analysis of annealed pure and composite Au-(TiO_2_, WO_3_), Ag-(TiO_2_, WO_3_) and Ag/Au-(TiO_2_, WO_3_) compositions are shown in Figure S7 of SI. As can be seen, at 633 nm excitation the intensity of defect peak (624 cm^−1^ for AgAu-TiO_2_ and 910 cm^−1^ for Ag/Au-WO_3_) in composites with bimetallic Ag/Au NPs is lower than that in the composites with Au NPs. 

**Figure 9:**
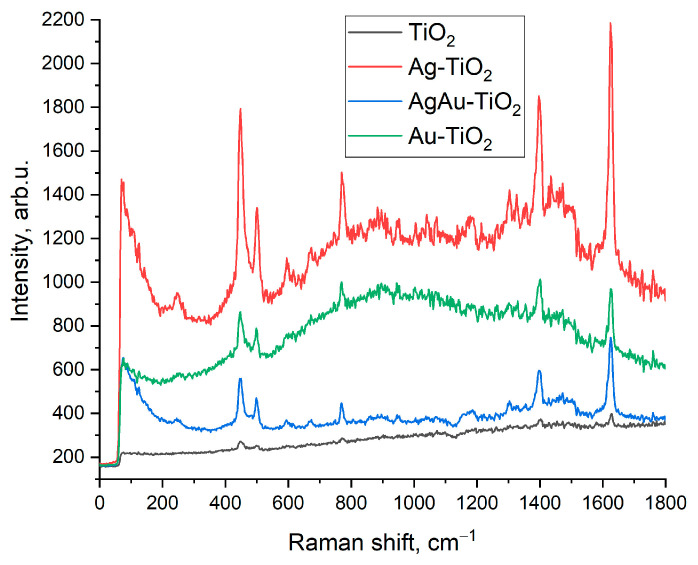
Comparison of Raman signal intensities of MB on pure TiO_2_ and composite Ag/Au-TiO_2_ substrates.

In general, effects considered in the work serve as an explanation of structural peculiarities of the metal-oxide systems under study and can be useful for understanding some features of defect regions at interfaces. From the practical point of view, considered composite films can be used, for example, as SERS substrates and refractive index detectors owing to the presence of metal nanoparticles on the surface. Figure 9 shows the results of the comparative analysis of SERS signals of Methylene blue dye (10^−5^ M) using films based on TiO_2_ with Ag, Ag/Au, and Au NPs. It was revealed that the most intensive Raman signal was detected using Ag-TiO_2_ substrate, as it was mentioned above that Ag NPs demonstrate the best plasmon properties as compared to NPs of Au and AgAu alloy. 

## 5. Conclusions

This work reports the study of the structural characteristics of Au-TiO_2_ and Au-WO_3_ plasmonic composite thin films by means of Raman spectroscopy. The composite thin films obtained by RF magnetron sputtering have the Au NPs and exhibit the LSPR band in the visible spectrum. The novelty of the paper consists of the detailed investigation of structural properties of plasmonic composites using different wavelengths for Raman measurements. This approach made it possible to reveal some peculiarities of the interaction between light, plasmonic metal nanoparticles, and surrounding metal oxide matrices, in particular, the effect of the rise and disappearance of the “defect peaks” in Raman spectra of TiO_2_ and WO_3_ depending on the excitation wavelength was detected. We suppose that the appearance of the peaks at 700–720 cm^−1^ and 935 cm^−1^ for TiO_2_ and WO_3_ matrices, respectively, is induced by the polarization (due to the charge transfer) of the matrix’s defective regions close to the interface with gold NPs. Thus, these findings provide insights into nanocomposite behavior, especially their interaction with light, and have potential applications in tailoring material properties.

## Data Availability

Not applicable.

## Supplementary Materials

The following supporting information can be downloaded at: www.mdpi.com/article/10.3390/ma16206809/s1, Figure S1: Results of EDS analysis of annealed samples under the study;. Figure S2: AFM images of the samples; Figure S3: Contrast AFM images of Au-TiO_2_ (a) and Au-WO_3_ (b) thin films used for size distribution analysis; Figure S4: Real and Imaginary part of dielectric permittivity of bulk Ag and Au according to Johnson et al, 1972; Figure S5: Raman spectra of Au NPs coated with ultra-thin (<10 nm) TiO_2_ (a) and WO_3_ (b) thin films. Figure S6: PL spectra of annealed Ag/Au-TiO_2_ (a) and Ag/Au-WO_3_ (b) thin films; Figure S7: Raman spectra of annealed pure and composite Ag/Au-TiO_2_ (a,b) and Ag/Au-WO_3_ (c,d) thin films acquired using 473 nm (a,c) and 633 nm (b,d) excitation lasers.

## References

[1] Quinten M. (2011). Optical Properties of Nanoparticle Systems: Mie and Beyond.

[2] Bohren C.F. (1983). Absorption and Scattering of Light by Small Particles.

[3] Messinger B.J., Von Raben K.U., Chang R.K., Barber P.W. (1981). Local Fields at the Surface of Noble-Metal Microspheres. Phys. Rev. B.

[4] Pilot R., Signorini R., Durante C., Orian L., Bhamidipati M., Fabris L. (2019). A Review on Surface-Enhanced Raman Scattering. Biosensors.

[5] Mayer K.M., Hafner J.H. (2011). Localized Surface Plasmon Resonance Sensors. Chem. Rev..

[6] Khurana K., Jaggi N. (2021). Localized Surface Plasmonic Properties of Au and Ag Nanoparticles for Sensors: A Review. Plasmonics.

[7] Guglielmelli A., Pierini F., Tabiryan N., Umeton C., Bunning T.J., De Sio L. (2021). Thermoplasmonics with Gold Nanoparticles: A New Weapon in Modern Optics and Biomedicine. Adv. Photonics Res..

[8] Baffou G., Cichos F., Quidant R. (2020). Applications and Challenges of Thermoplasmonics. Nat. Mater..

[9] Chehadi Z., Girardon J.S., Capron M., Dumeignil F., Jradi S. (2019). Thermoplasmonic-Induced Energy-Efficient Catalytic Oxidation of Glycerol over Gold Supported Catalysts Using Visible Light at Ambient Temperature. Appl. Catal. A Gen..

[10] Nakata K., Fujishima A. (2012). TiO_2_ Photocatalysis: Design and Applications. J. Photochem. Photobiol. C Photochem. Rev..

[11] Dahl M., Liu Y., Yin Y. (2014). Composite Titanium Dioxide Nanomaterials. Chem. Rev..

[12] Liu B., Wang J., Yang J., Zhao X. (2019). Charge Carrier Interfacial Transfer Pathways from TiO_2_ and Au/TiO_2_ Nanorod Arrays to Electrolyte and the Association with Photocatalysis. Appl. Surf. Sci..

[13] Tian Y., Tatsuma T. (2005). Mechanisms and Applications of Plasmon-Induced Charge Separation at TiO_2_ Films Loaded with Gold Nanoparticles. J. Am. Chem. Soc..

[14] Liu Z., Hou W., Pavaskar P., Aykol M., Cronin S.B. (2011). Plasmon Resonant Enhancement of Photocatalytic Water Splitting under Visible Illumination. Nano Lett..

[15] Panayotov D.A., Frenkel A.I., Morris J.R. (2017). Catalysis and Photocatalysis by Nanoscale Au/TiO_2_: Perspectives for Renewable Energy. ACS Energy Lett..

[16] Sarkar S., Gupta V., Tsuda T., Gour J., Singh A., Aftenieva O., Steiner A.M., Hoffmann M., Kumar S., Fery A. (2021). Plasmonic Charge Transfers in Large-Scale Metallic and Colloidal Photonic Crystal Slabs. Adv. Funct. Mater..

[17] Gupta V., Sarkar S., Aftenieva O., Tsuda T., Kumar L., Schletz D., Schultz J., Kiriy A., Fery A., Vogel N. (2021). Nanoimprint Lithography Facilitated Plasmonic-Photonic Coupling for Enhanced Photoconductivity and Photocatalysis. Adv. Funct. Mater..

[18] Ghosh A.K., Sarkar S., Tsuda T., Chae S., Knapp A., Nitschke M., Das A., Wießner S., König T.A.F., Fery A. (2023). Plasmonic Photoresistor Based on Interconnected Metal-Semiconductor Grating. Adv. Funct. Mater..

[19] An F.H., Yuan Y.Z., Liu J.Q., He M.D., Zhang B. (2023). Enhanced Electrochromic Properties of WO_3_/ITO Nanocomposite Smart Windows. RSC Adv..

[20] Guo J., Guo X., Sun H., Xie Y., Diao X., Wang M., Zeng X., Zhang Z. (2021). Bin Unprecedented Electrochromic Stability of A-WO_3-X_ Thin Films Achieved by Using a Hybrid-Cationic Electrolyte. ACS Appl. Mater. Interfaces.

[21] Brognara A., Bricchi B.R., William L., Brinza O., Konstantakopoulou M., Bassi A.L., Ghidelli M., Lidgi-Guigui N. (2022). New Mechanism for Long Photo-Induced Enhanced Raman Spectroscopy in Au Nanoparticles Embedded in TiO_2_. Small.

[22] Ye J., Arul R., Nieuwoudt M.K., Dong J., Zhang T., Dai L., Greenham N.C., Rao A., Hoye R.L.Z., Gao W. (2023). Understanding the Chemical Mechanism behind Photoinduced Enhanced Raman Spectroscopy. J. Phys. Chem. Lett..

[23] Markhabayeva A., Abdullin K., Kalkozova Z., Nurbolat S., Nuraje N. (2021). Effect of Synthesis Method Parameters on the Photocatalytic Activity of Tungsten Oxide Nanoplates. AIP Adv..

[24] Liang Y., Sun S., Deng T., Ding H., Chen W., Chen Y. (2018). The Preparation of TiO_2_ Film by the Sol-Gel Method and Evaluation of Its Self-Cleaning Property. Materials.

[25] Tehrani F.S., Ahmadian H., Aliannezhadi M. (2020). Hydrothermal Synthesis and Characterization of WO_3_ Nanostructures: Effect of Reaction Time. Mater. Res. Express.

[26] Lertthanaphol N., Pienutsa N., Chusri K., Sornsuchat T., Chanthara P., Seeharaj P., Kim-Lohsoontorn P., Srinives S. (2021). One-Step Hydrothermal Synthesis of Precious Metal-Doped Titanium Dioxide–Graphene Oxide Composites for Photocatalytic Conversion of CO_2_ to Ethanol. ACS Omega.

[27] Daughtry J., Alotabi A.S., Howard-Fabretto L., Andersson G.G. (2021). Composition and Properties of RF-Sputter Deposited Titanium Dioxide Thin Films. Nanoscale Adv..

[28] Cruz M.R.A., Sanchez-Martinez D., Torres-Martínez L.M. (2019). Optical Properties of TiO_2_ Thin Films Deposited by DC Sputtering and Their Photocatalytic Performance in Photoinduced Process. Int. J. Hydrogen Energy.

[29] Limwichean S., Kasayapanand N., Ponchio C., Nakajima H., Patthanasettakul V., Eiamchai P., Meng G., Horprathum M. (2021). Morphology-Controlled Fabrication of Nanostructured WO_3_ Thin Films by Magnetron Sputtering with Glancing Angle Deposition for Enhanced Efficiency Photo-Electrochemical Water Splitting. Ceram. Int..

[30] Torrell M., Adochite R.C., Cunha L., Barradas N.P., Alves E., Beaufort M.F., Rivière J.P., Cavaleiro A., Dosta S., Vaz F. (2012). Surface Plasmon Resonance Effect on the Optical Properties of TiO_2_ Doped by Noble Metals Nanoparticles. J. Nano Res..

[31] Alejandro L., Rivera-muñoz E.M., Rafael R., Escobar-alarc L., Esquivel K. (2021). Au-Ag/TiO_2_ Thin Films Preparation by Laser Ablation and Electrochemical Advanced Oxidation Processes (EAOP). Catalysts.

[32] Prikhodko O., Dosseke U., Nemkayeva R., Rofman O., Guseinov N., Mukhametkarimov Y. (2022). Localized Surface Plasmon Resonance Phenomenon in Ag/Au-WO_3-x_ Nanocomposite Thin Films. Thin Solid Films.

[33] Yergaliyeva S., Nemkayeva R., Guseinov N., Prikhodko O., Arbuz A., Orynbay B., Sagidolda Y., Aitzhanov M., Ismailova G., Mukhametkarimov Y. (2023). Synthesis and Optical Properties of Ag/Au-TiO_2_ Plasmonic Composite Thin Films. Opt. Mater. Express.

[34] Lyu P., Espinoza R., Nguyen S.C. (2023). Photocatalysis of Metallic Nanoparticles: Interband vs Intraband Induced Mechanisms. J. Phys. Chem. C.

[35] Souri A.P., Andrigiannaki N., Moschogiannaki M., Faka V., Kiriakidis G., Malankowska A., Zaleska-Medynska A., Binas V. (2021). Metal Titanate (ATiO_3_, A: Ni, Co, Mg, Zn) Nanorods for Toluene Photooxidation under Led Illumination. Appl. Sci..

[36] Bose R.J., Kavitha V.S., Sudarsanakumar C., Pillai V.P.M. (2016). Phase Modification and Surface Plasmon Resonance of Au/WO_3_ System. Appl. Surf. Sci..

[37] Jian A., Feng K., Jia H., Zhang Q., Sang S., Zhang X. (2019). Quantitative Investigation of Plasmonic Hot-Electron Injection by KPFM. Appl. Surf. Sci..

[38] Johnson P.B., Christy R.W. (1972). Optical Constants of the Noble Metals. Phys. Rev. B..

