# Supplementary material for: Peculiarities of the Structure of Au-TiO2 and Au-WO3 Plasmonic Nanocomposites
Source: Materials (Basel). 2023 Oct 22;16(20):6809. doi: 10.3390/ma16206809 (PMC10608088; doi:10.3390/ma16206809)
Supplement: Supplementary file 1 [file materials-16-06809-s001.zip › materials-2645646-supplementary.pdf]

materials

MDPI

Supplementary Information

Peculiarities of Structure of Au-TiO_2 and Au-WO_3 Plasmonic Nanocomposites

Yerulan Sagidolda [1,2], Saule Yergaliyeva [1], Zhandos Tolepov [1,2], Guzal Ismailova [1], Bakytzhan Orynbay [1,2], Renata Nemkayeva [2], Oleg Prikhodko [1], Svetlana Peshaya [1], Suyumbika Maksimova [1], Nazim Guseinov [2] and Yerzhan Mukhametkarimov [1,2,*]

[1] Department of Physics and Technology, Al-Farabi Kazakh National University, Al-Farabi av. 71, 050040 Almaty, Kazakhstan; erulan.sagidolda@kaznu.kz (Y.S.); oleg.prikhodko@kaznu.kz (O.P.); svetlana.mikhailova@kaznu.kz (S.P.);
[2] National Nanotechnology Laboratory of Open Type, Al-Farabi av. 71/23, 050040 Almaty, Kazakhstan
* Correspondence: yerzhan.mukhametkarimov@kaznu.edu.kz

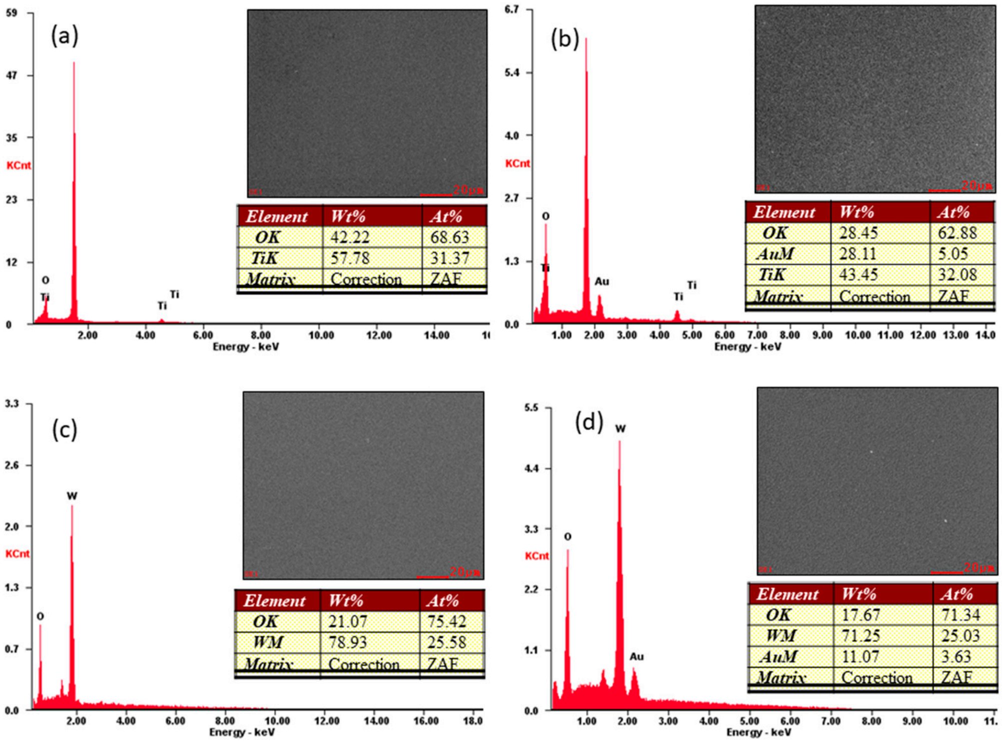

Figure S1. Results of EDS analysis of annealed samples under the study (a) TiO_2; (b) Au-TiO_2; (c) WO_3; (d) Au-WO_3.

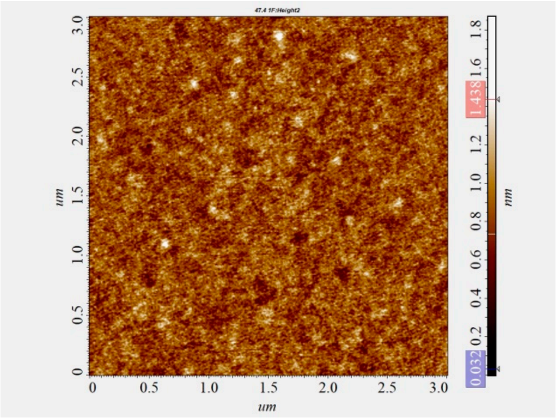

a) AFM image of as-deposited TiO_2 thin film

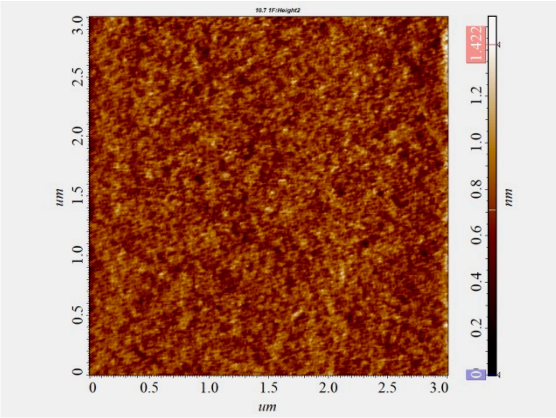

b) AFM image of as-deposited WO_3 thin film

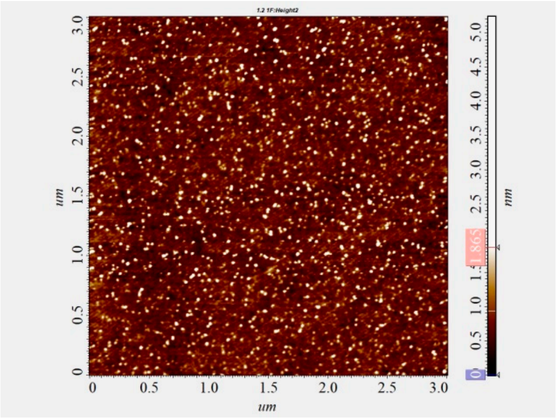

c) AFM image of as-deposited Au-TiO_2 thin film

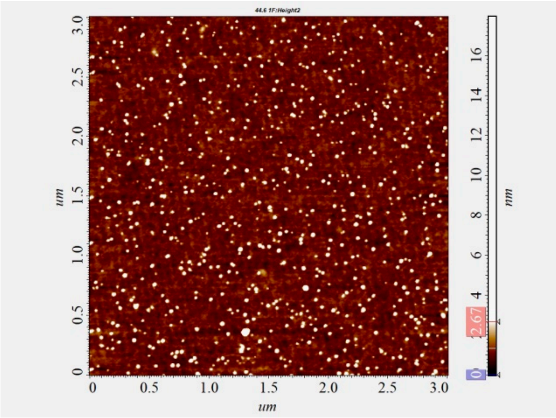

d) AFM image of as-deposited Au-WO_3 thin film

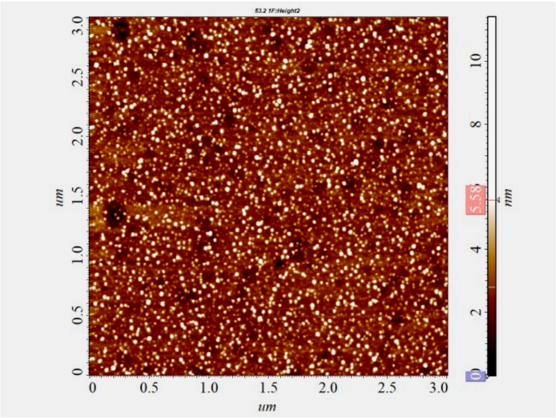

e) AFM image of annealed Au-TiO_2 thin film

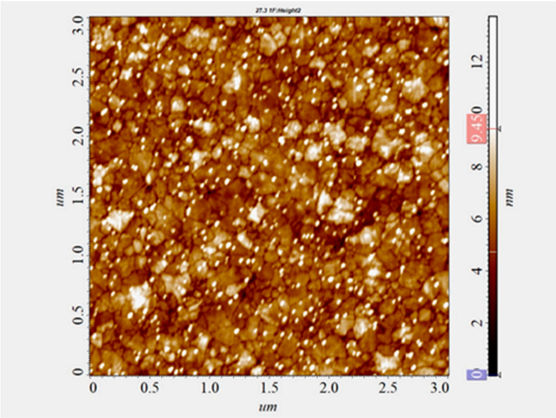

f) AFM image of annealed Au-WO_3 thin film

Figure S2. AFM images of the samples.

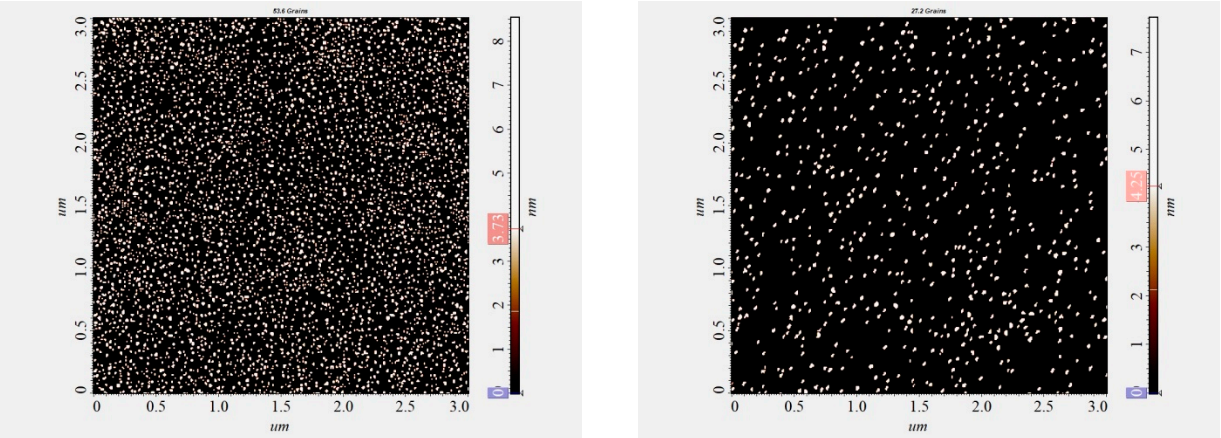

(a) (b)

Figure S3. Contrast AFM images of Au-TiO_2 (a) and Au-WO_3 (b) thin films used for size distribution analysis.

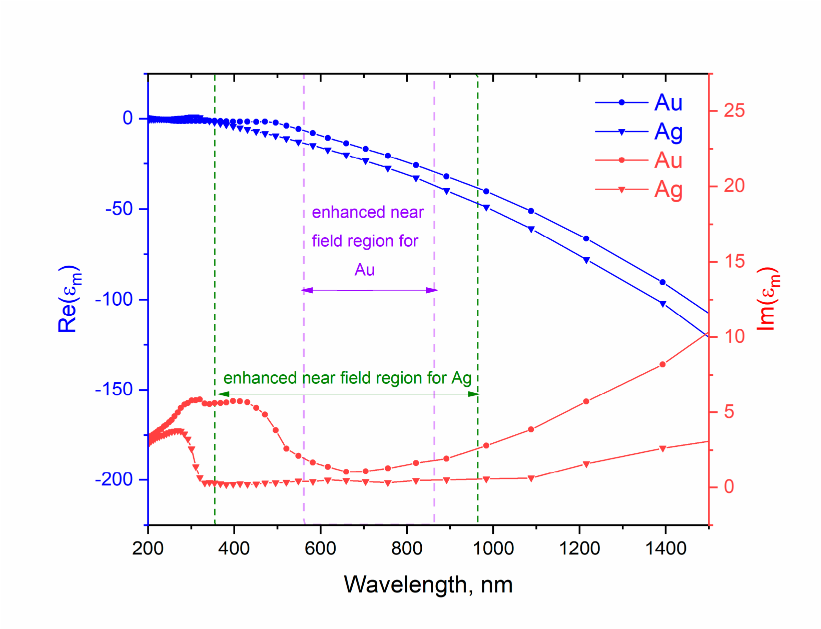

Figure S4. Real and Imaginary part of dielectric permittivity of bulk Ag and Au according to Johnson et al, 1972.

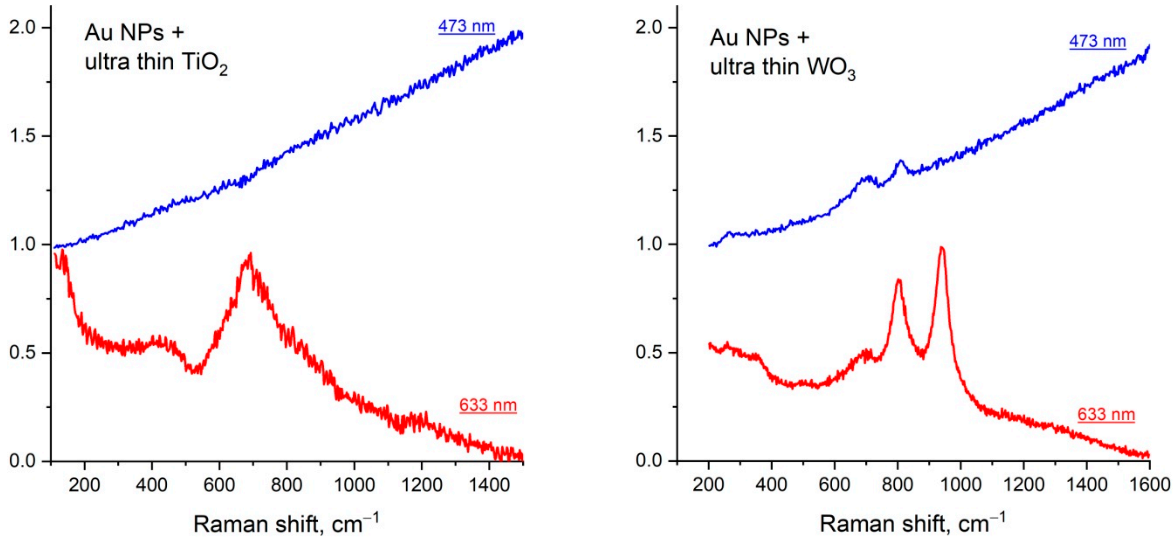

a) **b)**

Figure S5. Raman spectra of Au NPs coated with ultra-thin (<10 nm) TiO_2 (a) and WO_3 (b) thin films.

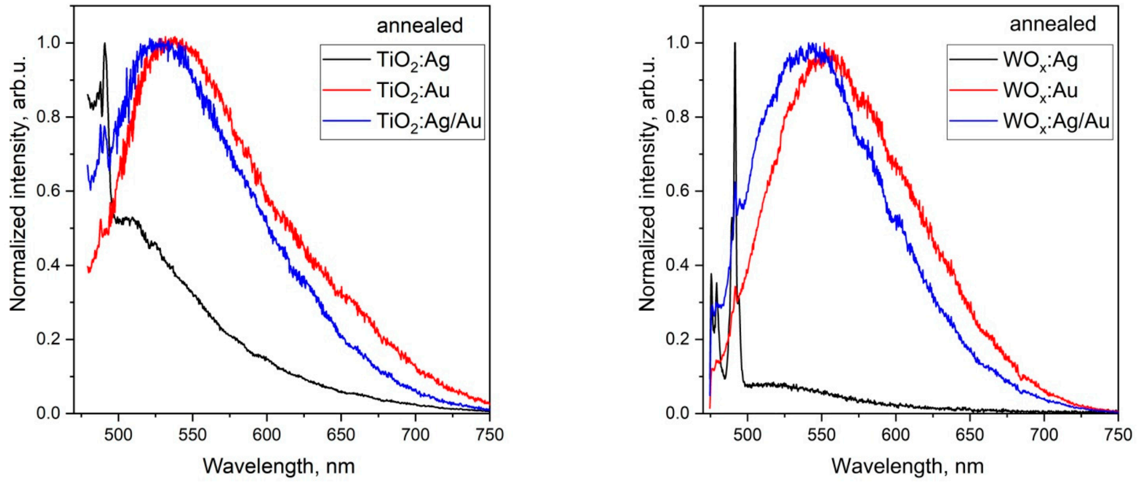

a) **b)**

Figure S6. PL spectra of annealed Ag/Au-TiO_2 (a) and Ag/Au-WO_3 (b) thin films.

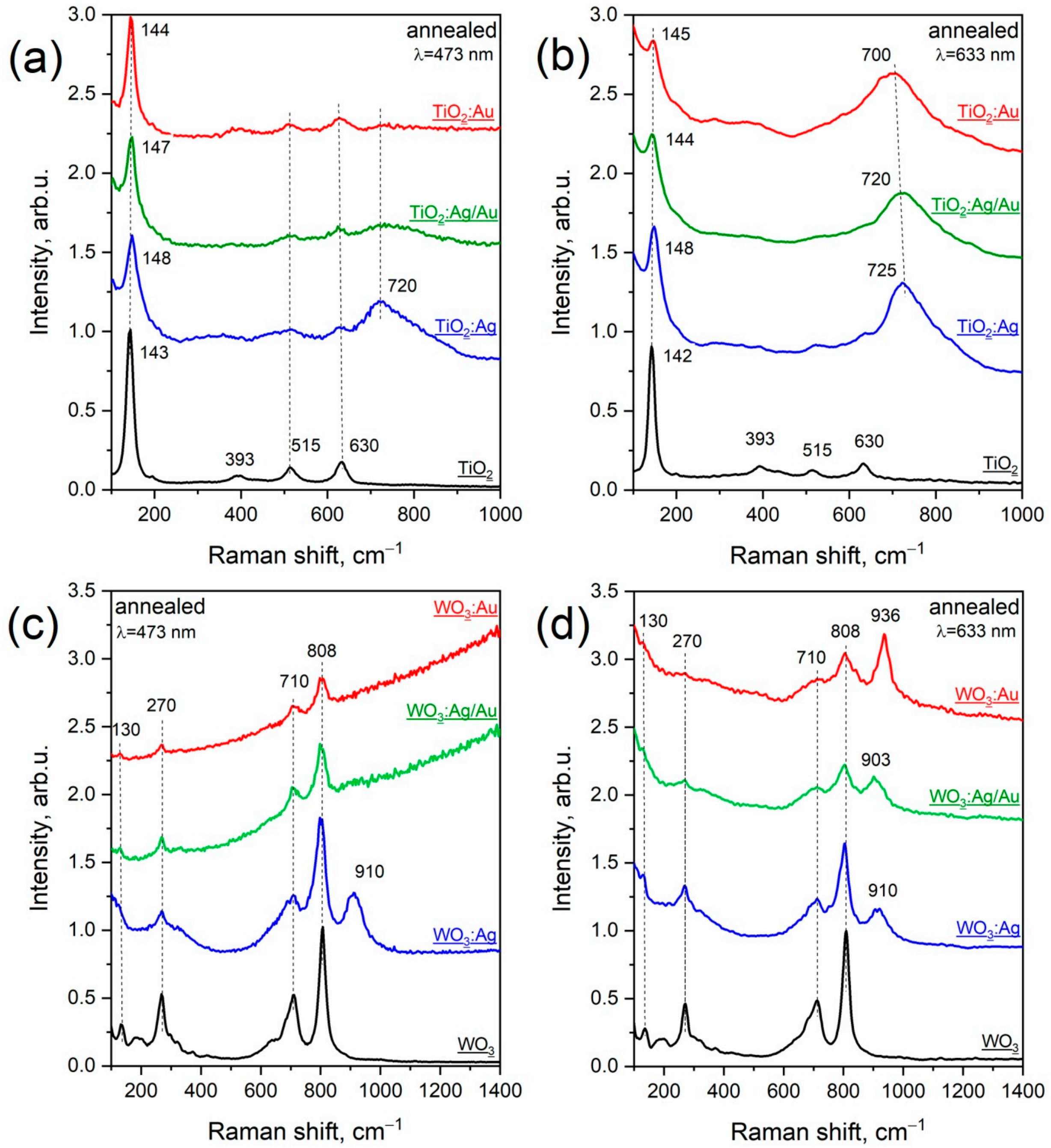

Figure S7. Raman spectra of annealed pure and composite Ag/Au-TiO_2 (a,b) and Ag/Au-WO_3 (c,d) thin films acquired using 473 nm (a,c) and 633 nm (b,d) excitation lasers.
